# A Simple and Novel Method to Monitor Breathing and Heart Rate in Awake and Urethane-Anesthetized Newborn Rodents

**DOI:** 10.1371/journal.pone.0062628

**Published:** 2013-05-03

**Authors:** Christoph M. Zehendner, Heiko J. Luhmann, Jenq-Wei Yang

**Affiliations:** Institute of Physiology and Pathophysiology, University Medical Center of the Johannes Gutenberg-University, Mainz, Germany; Scuola Superiore Sant'Anna, Italy

## Abstract

Rodents are most useful models to study physiological and pathophysiological processes in early development, because they are born in a relatively immature state. However, only few techniques are available to monitor non-invasively heart frequency and respiratory rate in neonatal rodents without restraining or hindering access to the animal. Here we describe experimental procedures that allow monitoring of heart frequency by electrocardiography (ECG) and breathing rate with a piezoelectric transducer (PZT) element without hindering access to the animal. These techniques can be easily installed and are used in the present study in unrestrained awake and anesthetized neonatal C57/Bl6 mice and Wistar rats between postnatal day 0 and 7. In line with previous reports from awake rodents we demonstrate that heart rate in rats and mice increases during the first postnatal week. Respiratory frequency did not differ between both species, but heart rate was significantly higher in mice than in rats. Further our data indicate that urethane, an agent that is widely used for anesthesia, induces a hypoventilation in neonates whilst heart rate remains unaffected at a dose of 1 g per kg body weight. Of note, hypoventilation induced by urethane was not detected in rats at postnatal 0/1. To verify the detected hypoventilation we performed blood gas analyses. We detected a respiratory acidosis reflected by a lower pH and elevated level in CO_2_ tension (pCO_2_) in both species upon urethane treatment. Furthermore we found that metabolism of urethane is different in P0/1 mice and rats and between P0/1 and P6/7 in both species. Our findings underline the usefulness of monitoring basic cardio-respiratory parameters in neonates during anesthesia. In addition our study gives information on developmental changes in heart and breathing frequency in newborn mice and rats and the effects of urethane in both species during the first postnatal week.

## Introduction

Brain function in mammals is strongly dependent upon intact neurovascular coupling [1]. Most *in vivo* studies investigating neuronal properties require anesthesia [2], [3]. However, neurovascular coupling may be impaired by anesthetic procedures [2], [3]. It has been recently shown that anesthetics are capable of modulating cerebrovascular autoregulation and blood-brain barrier integrity under various circumstances [4]–[6]. It is further known that anesthetic agents like urethane lower pH, induce alterations in the respiratory rate of adult rodents [7] and have an effect on spontaneous activity patterns in the cortex [8].

Thus it is important to control basic physiological parameters such as body temperature, breathing rate and heart frequency to minimize experimental errors in *in vivo* studies due to anesthesia.

Various techniques have been described to monitor breathing and heart rate in rodents [9]–[12]. An established technique to record ECG and breathing in newborn mice and rats is available using plethysmography and ECG electrodes imbedded in a sealed chamber in which the animals are placed [9], [11]. This method is quite elegant as it is non-invasive and gives the opportunity to detect tidal volumes. However this approach does not allow to record ECG and breathing while performing experimental manipulation at the same time due to the sealed chamber in which the animal is placed for plethysmography. Therefore these techniques have limitations [10] and it remains a difficult task to measure cardiovascular parameters in unrestrained newborn animals. Hence we sought to develop a method that allows access to the animal while recording cardio-respiratory parameters. Here we describe an easy technique to record breathing and heart rate in awake and anesthetized newborn C57/Bl6 mice and Wistar rats. An advantage of the presented setup is the possibility to do experimental manipulations while recording cardio-respiratory parameters.

## Materials and Methods

### Rodents, Ethics Statement

Neonatal C57/Bl6 mice and Wistar rats at the age of P0/1 and P6/7 obtained by vaginal delivery were examined. All procedures were performed in accordance with European laws (86/609/EEC) and national laws on animal handling. Great care was taken to minimize the number of animals and their suffering. Animals were taken care of in compliance with institutional guidelines of the University Medical Center of the Johannes Gutenberg-University, Mainz. The Animal Ethics Committee of the “Landesuntersuchungsamt Rheinland-Pfalz” and the authorities of the “Landesuntersuchungsamt Rheinland-Pfalz” approved all experiments, protocol number: “Aktenzeichen 23 177-07/G 10-1-010 and 23 177-07/G 12-1-070”.

### ECG

An electrocardiographic signal was obtained from the neonatal rodents by a two channel ECG. For this purpose soft electrodes were gently attached at the right front paw and at the tail or the left hind limb. Conductance of the electrical signal evoked by the cardiac vector was increased by electrode gel (Parkland laboratories, New Jersey, USA). Pups were placed on a 37°C heating plate. Cotton was placed around the pups to make them feel comfortable and body temperature was monitored with a thermal probe (Oxylite 4000, Oxford Optronix) which was placed in the interscapular space similar as described elsewhere [9]. Animals were allowed to adapt to the new environment for at least 5 minutes. Subsequently recordings were performed for a minimum of 5 minutes. ECG was recorded at a sampling rate of 20 kHz using Spike2 or ME-Systems (Multi Channel Systems, Reutlingen, Germany) without any amplification. Data were analyzed offline using MATLAB software version 7.7 (MathWorks). Heart rate data were analyzed after band pass filtering (10-200 Hz, Butterworth three-order filter). Heart beats per minute (BPM) were determined by analyzing R to R intervals after visual identification of QRS waves. At least 3 calculations from each animal were averaged over a period of 3 seconds recording or longer. BPM were obtained before (awake) and after urethane administration.

### Piezoelectric Transducer (PZT) Recordings

A PZT element (disk shape, 3.6 cm outer diameter, FT-Serie 30 V/AC 2.6±0.3 kHz) was used for the breathing recording. Pups were placed on a temperature monitored heating pad, surrounded by soft cotton and were allowed to adapt to the new environment for at least 5 minutes as described above. Beneath the animals a PZT element was placed. Piezoelectric signals were collected at a sampling rate of 20 kHz using Spike2 or ME-Systems without any amplification and further analyzed offline using MATLAB software version 7.7. RPM was measured by counting breathing cycles per second followed by multiplication with factor 60: RPM = [breathing cycles per second] × 60.

For calculation of relative changes in RPM all RPM values were set in relation to the average RPM of non-anesthetized animals. Subsequently relative RPM in percentage of control were obtained. Next relative reduction in RPM was determined by the following formula: RPM[% non- anesthetized] - RPM[% urethane]. RPM was obtained before (awake) and after urethane administration. During all recordings in this study body temperature was maintained at about 37°C by a heating pad and monitored with a thermal probe as indicated above.

### Urethane Administration

Urethane (from Sigma-Aldrich, Taufkirchen, Germany) was injected intraperitoneally (i.p.) at a dose of 1 g per kg body weight. Injection volumes for mice and rats were 6.67 µl per g body weight. For this purpose luer tip Hamilton syringes (Hamilton Company ordered via Sigma) were used. For blood gas analyses sham animals received Ringer (Braun, Melsungen, Germany) solution i.p. at equal volumes as urethane treated pups. Recordings were initiated 30–60 minutes after administration of urethane.

### Blood Gas Analyses

We analyzed pH, pCO_2_, hemoglobin, glucose, base excess in extracellular fluid and standard HCO_3_^−^ using a clinical blood gas analyzing device (ABL90 FLEX, Radiometer, Germany). Neonates were rapidly decapitated. Subsequently arterial/venous blood was carefully collected from the neck with a heparinized capillary. Great care was taken to analyze the probes immediately. Body temperature was held in a physiological range of about 37°C by a heating pad and monitored with a thermal probe as mentioned before.

### Ethanol Quantification in Blood Plasma

Anesthetized rodents were decapitated 60 minutes after urethane administration. Arterial/venous blood was collected from the neck with heparinized syringes immediately and stored on ice. Blood plasma was separated from cellular blood components by centrifugation for 10–15 minutes at 4°C, 2000 rounds per minute and subsequently stored at -20°C until further analyses were performed. Ethanol in blood plasma probes was analyzed using an ethanol assay kit as recommended by the manufacturer (Abcam, Cambridge, UK). 10 µl of blood plasma were incubated for 30 minutes with the assay kit reaction mixture at 37°C in black 96 Well plates (Greiner, ordered via Sigma-Aldrich, Taufkirchen, Germany). Fluorescent signals were detected in an infinite M1000 Tecan Plate reader equipped with i-control 6 software (Tecan Trading AG, Männderdorf, Switzerland). Excitation was 535 nm, emission was detected at 587 nm. Ethanol concentration in µmol/l was calculated as recommended by the manufacturer’s instructions.

### Statistics

Results are documented as mean ± standard deviation (SD). For data presentation box and whisker plots are shown, whiskers indicate minimum and maximum values. Data were analyzed using Graphpad Prism 4 version 4.02 for windows. All datasets passed the D’Agostino and Pearson omnibus normality test and were therefore analyzed by t-test. Cardio-respiratory parameters were obtained before (awake) and after urethane administration. Therefore paired t-tests were used to evaluate the effect of urethane on breathing and heart rate. In all other comparisons an unpaired t-test was used. Differences of values were considered to be statistically significant at P<0.05.

## Results

### Experimental Setup and Recordings

**Figure 1:**
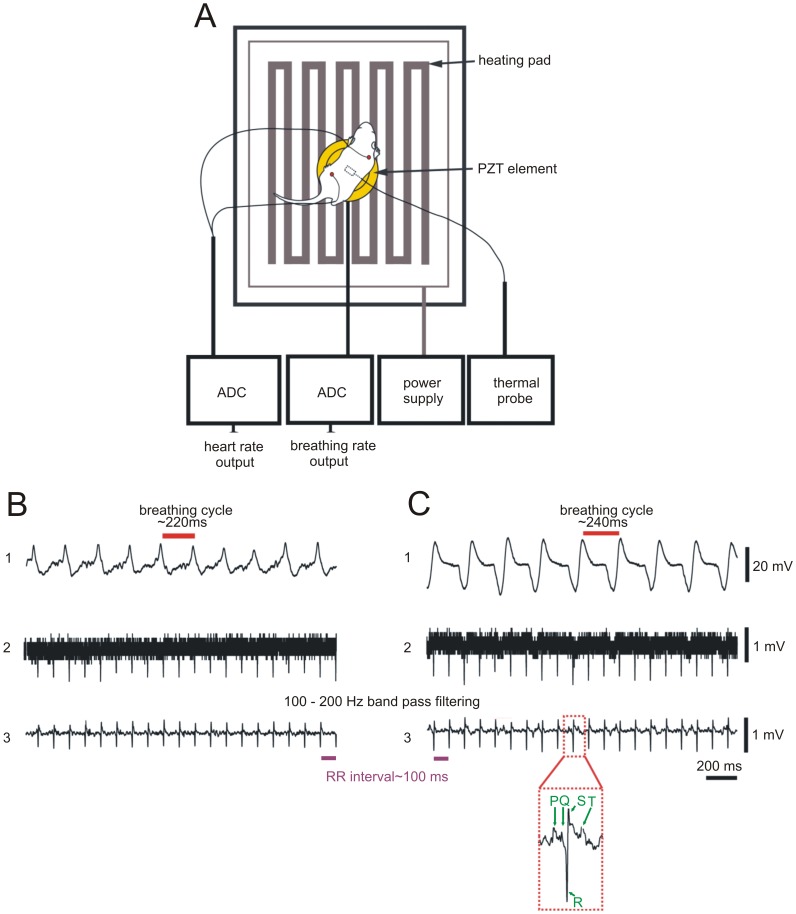
Monitoring setup, ECG and breathing detection. Rodents were placed on a temperature monitored heating pad and connected to ECG electrodes. A thermal probe was used to control the body temperature of the animal. Data of heart and breathing rates were collected with analog digital converters (ADC, **A**). Recordings from an awake P7 mouse showing a raw data trace of breathing movements including breathing cycle duration (**B, trace 1**). Heart rate recordings in awake animals (raw data: **B,** **trace 2**) were filtered at 10–200 Hz band pass which allowed identification of QRS complexes (**B,** **trace 3**). Effect of urethane on breathing and heart rate in representative recordings of the same animal 30–60 minutes after urethane administration (**C**). Note the different pattern of the PZT signal (**C, trace 1**) in comparison with the awake state in B trace 1. After filtering the ECG raw data (**C, trace 2**) in some recordings beside QRS complexes P and T waves could be identified (**C, trace 3 inset**).

Mice or rats were placed on a PZT element equipped with a temperature monitored heating pad. Body temperature was monitored by a thermal probe and maintained at 37°C. Two soft electrodes were attached to the right paw and the left hind limb or tail (**Figure 1** **A**). We recorded breathing in awake animals by detection of movements of the thorax or abdomen (**Figure 1** **B, trace 1)** with the help of the PZT. Via electrocardiographic recording we determined heart frequency in the awake animals (**Figure 1** **B, traces 2 and 3)**. Similar results were obtained in urethane anesthetized animals (**Figure 1** **C**). After 10 to 200 Hz band pass filtering of the ECG raw data (**Figure 1** **B, C, trace 2**) QRS complexes could be better identified (**Figure 1** **B, C, trace 3**). In some recordings a P-wave followed by a QRS complex and a T wave could be detected (**Figure 1** **C, inset**).

### Changes in Cardio-respiratory Physiology during the First Postnatal Week

**Figure 2:**
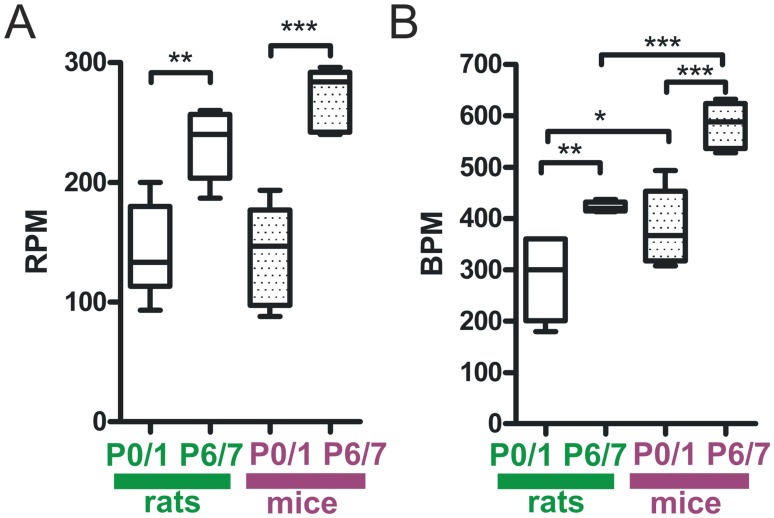
Developmental changes in respiratory and heart rate during the first postnatal week. In rats and mice breathing frequency significantly increased during the first postnatal week (**A**). We did not observe differences in RPM at corresponding ages between mice and rats. Heart rate increased from P0/1 to P6/7 in both species (**B**). Note that mice have significantly faster heart frequencies than rats at the same age. Box and whisker plots (displaying 75th percentile, median and 25th percentile) are shown, whiskers indicate minimum and maximum values. *P<0.05, **P<0.01, ***P<0.001.

To determine developmental changes in awake unrestrained mice and rats in cardio-respiratory physiology during the first neonatal week, we evaluated heart and breathing rate at postnatal day 0/1 (P0/1) and at P6/7. Breathing and heart rate increased significantly within the first 7 postnatal days in both species. Respiration rate in P0/1 rats was significantly lower compared to P6/7 (rats P0/1∶144±39 respirations per minute (RPM) vs. rats P6/7∶232±30 RPM at P6/7, n = 5–7 animals, P<0.01, **Figure 2** **A**). In mice we obtained similar data (mice P0/1∶140±38 RPM vs. mice P6/7∶270±26 RPM, n = 5–6 animals, P<0.001, **Figure 2** **A**). No significant differences in RPM were noted between rats and mice of the same age.

Heart frequency in P0/1 mice and rats increased after birth (rats P0/1∶276±74 beats per minute (BPM) vs. rats P6/7∶423±9 BPM, n = 5–7 animals, P<0.01; mice P0/1∶379±67 BPM vs. mice P6/7∶582±45 BPM, n = 5–6 animals, P<0.001, **Figure 2** **B**). Heart frequency of rats was significantly slower compared to mice of the same age (rats P0/1∶276±74 BPM vs. mice P0/1∶379±67 BPM, n = 6–7 animals, P<0.05; rats P6/7∶423±9 BPM vs. mice P6/7∶582±45 BPM, n = 5 animals, P<0.001, **Figure 2** **B**).

### Influence of Urethane on Breathing Frequency and Heart Rate in Newborn Rodents

**Figure 3:**
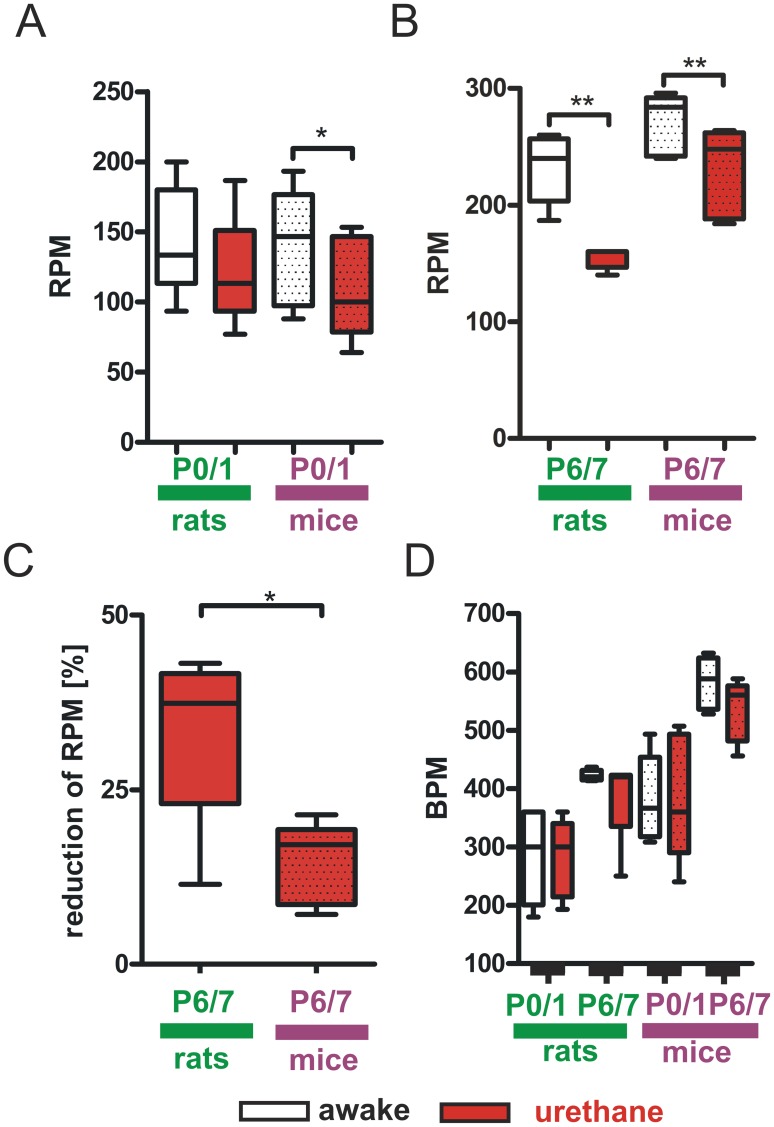
Impact of urethane anesthesia on cardio-respiratory parameters. In P0/1 mice RPM is significantly reduced by urethane but not in P0/1 rats (**A**). At P6/7 urethane resulted in a significant decrease of RPM in both species (**B**). The reduction of RPM was significantly higher in rats compared to mice (**C**). Urethane had no significant impact on heart rates in both species at all ages (**D**). Box and whisker plots (displaying 75th percentile, median and 25th percentile) are shown, whiskers indicate minimum and maximum values. *P<0.05, **P<0.01.

Urethane is widely used to induce anesthesia [13]. Therefore we elucidated its effect on heart and respiratory rate at a dose of 1 g per kg body weight 30 to 60 minutes after its administration. This dosage is commonly used for anesthetic purposes in rodents [14]. At P0/1 we observed a significant reduction of RPM in urethane treated mice compared with the awake state (mice P0/1 awake: 140±38 RPM vs. mice P0/1 urethane: 108±33 RPM, n = 6 animals, P<0.05, **Figure 3** **A**). RPM in P0/1 rats was not significantly reduced upon urethane treatment (rats P0/1 awake: 144±39 RPM vs. rats P0/1 urethane: 120±38 RPM, n = 7 animals, P>0.05, **Figure 3** **A**). At P6/7 urethane reduced RPM in both species (rats P6/7 awake: 232±30 RPM vs. rats P6/7 urethane: 155±9 RPM, n = 5 animals, P<0.01; mice P6/7 awake: 270±26 RPM vs. mice P6/7 urethane: 230±39 RPM, n = 5 animals, P<0.01, **Figure 3** **B**). To determine if the urethane induced depression of breathing was more severe in one of the two species we evaluated the relative reduction of RPM in both groups at P6/7. We found that breathing frequency of P6/7 rats was significantly more diminished by urethane than in P6/7 mice (rats P6/7 urethane: 33±13% vs. mice P6/7 urethane: 15±6%, n = 5 animals, P<0.05, **Figure 3** **C**). Contrary to our findings on the effect of urethane on breathing, heart rate did not change significantly upon urethane administration (rats P0/1 awake: 276±74 BPM vs. rats P0/1 urethane: 281±65 BPM, n = 7 animals; rats P6/7 awake: 423±9 BPM vs. rats P6/7 urethane: 387±77 BPM, n = 5 animals; mice P0/1 awake: 379±67 BPM vs. mice P0/1 urethane: 381±99 BPM, n = 6 animals; mice P6/7 awake: 582±45 BPM vs. mice P6/7 urethane: 535±53 BPM, n = 5 animals, P>0.05 in all groups, **Figure 3** **D**).

### Impact of Urethane on Acid-base Status of Neonatal Mice and Rats

**Figure 4:**
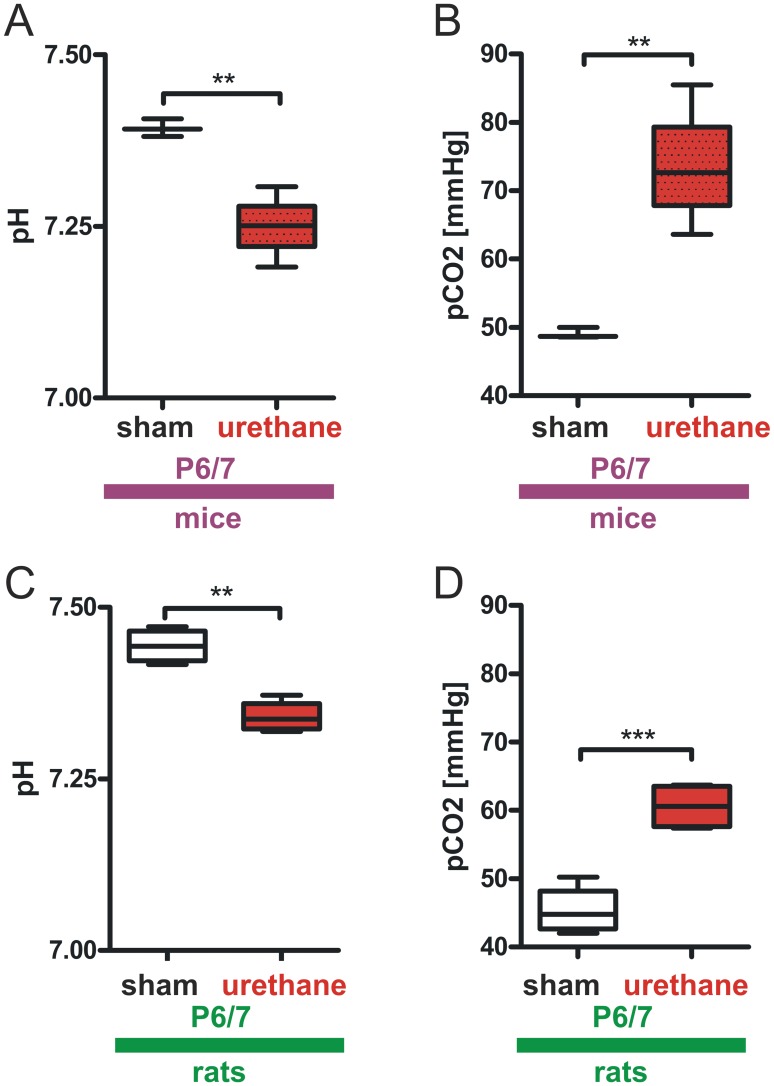
Urethane anesthesia results in respiratory acidosis in P6/7 mice and rats. Blood gas analyses revealed a significant reduction in pH during urethane-anesthesia in P6/7 mice (**A**) and elevated pCO_2_ levels (**B**). Similar values were obtained in rats. Here, urethane administration resulted in a diminished pH (**C**) and was accompanied by an increased pCO2 (**D**). Box and whisker plots (displaying 75th percentile, median and 25th percentile) are shown, whiskers indicate minimum and maximum values. **P<0.01, ***P<0.001.

In our study we measured thoracic or abdominal movements as parameters for breathing. It is known that excursions of the thorax and abdomen correlate to breathing frequency and activity of the phrenic nerve [15]. However, to strengthen the significance of our finding that RPM is reduced upon urethane administration we performed blood gas analyses. It is known that hypoventilation results in perturbances in acid-base status due to retention of CO_2_ [16]. Urethane lowered RPM in both mice and rats at P6/7. Thus we analyzed blood gases in this age group to evaluate if the detected reduction on RPM by the piezoelectric technique relates to a reduction in lung ventilation. We found that urethane significantly lowered pH and increased CO_2_ partial pressure in both species (mice sham P6/7∶7.39±0.01 pH vs. mice urethane P6/7∶7.25±0.05 pH; mice sham P6/7∶49.1±0.78 mmHg pCO_2_ vs. mice urethane P6/7∶73.58±9.02 mmHg pCO_2_, n = 3–4 animals, P<0.01 in all groups, **Figure 4** **A**, **B**, **Table 1**; rats sham P6/7∶7.44±0.03 pH vs. rats urethane P6/7∶7.34±0.02 pH, P<0.01; rats sham P6/7∶45.43±3.64 mmHg pCO_2_ vs. rats urethane P6/7∶60.58±3.39 mmHg pCO_2_, n = 4 animals, P<0.001, **Figure 4** **C**, **D**, **Table 2**). Base excess in extracellular fluid (BE_ECF_) and standard bicarbonate (HCO_3_^−^_standard_) levels did not significantly differ between sham and urethane treated animals (**Tables 1** **,** **2**). We detected an increase of blood glucose levels in both species upon urethane treatment, however this increase was significantly elevated only in rats but not in mice (**Tables 1** **,** **2**). Additional data on levels of hemoglobin, blood glucose and bodyweight are displayed in tables 1 and 2.

**Table 1:** Effect of urethane on blood gases in P6/7 mice.

	sham	urethane
**BE_ECF_** [mmol/l]	5±0.78	4.85±0.54
**HCO_3_** ^−^ **_standard_ [**mmol/l]	28.4±0.89	28.28±0.49
**Hemoglobin** [g/l]	84±9.5	87.25±10.69
**Glucose** [mg/dl]	161±13.1	176.8±18.1

Table 1: n = 3–4 animals, body weight [g] = 4.3±0.3, Values are mean ± SD, arterial/venous. blood.

**Table 2:** Effect of urethane on blood gases in P6/7 rats.

	sham	urethane
**BE_ECF_** [mmol/l]	6.98±0.66	7±1.1
**HCO_3_** ^−^ **_standard_ [**mmol/l]	29.5±0.54	29.23±1.05
**Hemoglobin** [g/l]	88.75±5.32	90±0.82
**Glucose** [mg/dl]	125.8±9.54	150±1.83**

Table 2: n = 3–4 animals, body weight [g] = 18.4±2.2; Values are mean ± SD, **P<0.01, arterial/venous blood.

### Determination of the Urethane Metabolite Ethanol in Blood Plasma of Mice and Rats

**Figure 5:**
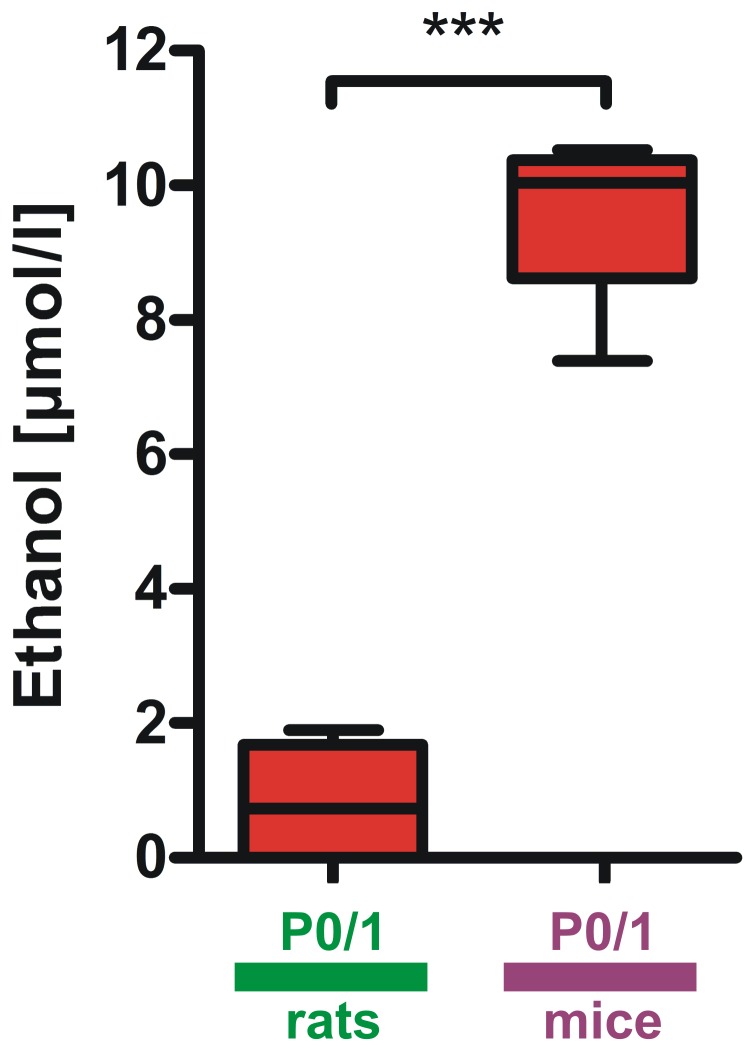
Differences of urethane metabolism in mice and rats within the first postnatal week. In P0/1 mice ethanol concentrations in blood plasma 60 minutes after urethane administration were significantly higher than in P0/1 rats. Ethanol in plasma from P6/7 rodents was not detected. Box and whisker plots (displaying 75th percentile, median and 25th percentile) are shown, whiskers indicate minimum and maximum values. ***P<0.001.

To elucidate if urethane metabolism differs between P0/1 and P6/7 we determined one of the major metabolites of urethane, ethanol [17], in blood plasma probes 60 minutes after urethane treatment. Ethanol concentration in P0/1 mice was significantly higher than in P0/1 rats (mice P0/1 9.5±1.43 µmol/l vs. P0/1 rats 0.84±0.99 µmol/l, n = 4 animals per group, P<0.001, **Figure 5**). Ethanol in plasma probes was not detectable at P6/7 in mice and rats (n = 4 animals per group).

## Discussion

In summary our study demonstrates that **i)** ECG and breathing can be detected in awake unrestrained newborn mice and rats with a rather simple technical approach; **ii)** breathing and heart rate increase within the first postnatal week in C57Bl6 mice and Wistar rats; **iii)** heart frequency of newborn C57/Bl6 mice is faster than in Wistar rats whilst breathing rate in both species is not different at corresponding ages; **iv)** the anesthetic agent urethane induces hypoventilation and consecutive respiratory acidosis in P6/7 mice and rats while heart rate remains unaffected within the first hour of administration. This urethane-induced hypoventilatory effect was also observed in P0/1 mice but not in P0/1 rats; **v**) Urethane pharmacokinetics are different in P0/1 mice and rats and between P0/1 and P6/7 in both rodent species.

The presented technical approach has the advantage that it is quite easy to install and cost-effective. It can be installed with commonly used analog digital converters. This allows to precisely correlate ECG and breathing recordings with other analog digital readings e.g. laser doppler flow investigations or electrophysiological measurements at the same time. Furthermore it is not necessary to restrain the newborn animals for recordings in our experimental setup. Because of the small size of the PZT element and the ECG electrodes it is feasible to easily integrate the setup into experimental procedures performed e.g. during stereotactical injections into the rodent brain or procedures involving craniotomies. Clearly a limitation of the technique is that the PZT element cannot directly detect tidal volumes of lung ventilation. This can be estimated by plethysmography [9]–[11]. However the presented blood gas analyses indicate that breathing frequency detected with the help of the PZT correlate to lung ventilation. One should reflect that the PZT element is very sensitive to movements. Any manipulation while breathing is recorded with the PZT may cause artifacts in the measurements.

In rodents it is well documented that breathing patterns and heart frequency significantly change under physiological as well as pathophysiological conditions during early development [10], . However, there are no reports regarding changes in respiration and heart frequency in awake versus urethane anesthetized newborn C57Bl6 mice and Wistar rats during the first postnatal week.

We were able to detect P waves, QRS complexes and T waves in newborn mice and rats with a two channel ECG. One may argue that the attachment of ECG electrodes may induce sympathetic distress and impair validity of recordings. Discomfort may lead to sympathetic nerve activation and results in release of stress hormones e.g. catecholamines that are able to affect heart frequency [16], [22]. However we did not observe signs of discomfort in rodent pups throughout the duration of experiments. Furthermore, our findings on heart rate increase in newborn rodents are in line with similar data reported previously by other groups [19] that made use of electrocardiography electrodes (non-attaching electrodes by paw contact) and piezoelectric techniques [10], [12], [23].

Previously it has been demonstrated that a piezoelectric element is capable of detecting heart beat and respiratory rate in anesthetized mice [24]. Here we show that this technique is further capable to determine breathing frequency in non-anesthetized newborn mice and rats. Our results also show an increase in respiration in both rodent species within the first postnatal week as obtained by piezoelectric recordings. In contrast, during human development basal heart rate drops from of about 80–180 BPM [25] in newborn babies to values below 100 BPM [16] in adults while respiratory frequency decreases from 30–60 RPM [25] in neonates to about 16 RPM [16] in adolescence and adulthood. Similar cardio-respiratory changes were observed in larger mammals such as lambs or pigs [26], [27]. Some data have demonstrated that an increase of body size as well as maturation correlates with a reduction of respiratory frequency in different mammalian species [15], [21]. However it has been shown that not only body weight or age but also genetic differences may cause variances in baseline respiratory frequency [28]. Our finding on an increase of breathing frequency in newborn rodents is further supported by datasets from various other research groups [10], [23].

One should be aware that physiological changes in small animals, such as mice and rats, significantly differ from larger mammals and may be crucially dependent on genetic background [28].

Interestingly Robinson et al. reported a high amount of apneic phases in P0 Swiss CD-1 mice in about 29% of the total breathing time within similar recording periods [10] and this value was significantly lower in older animals. In our experimental setting we did not observe such an amount of apneas in P0/1 C57/Bl6 mice or Wistar rats. It has been reported that breathing patterns in rodents are significantly dependent on genetic background [28]. Therefore differences in breathing patterns in newborn Swiss CD-1 and C57/Bl6 mice may be due to genetic differences in both mouse strains. However, in line with data by Robinson and coworkers we found that respiratory rate at P0/1 significantly increases until P6/7 in mice and rats [10].

We would like to emphasize that a technical limitation of the piezoelectric transducer technique is based on measuring excursions of the thorax or abdomen and can be disturbed by body movements which is also reflected by the clearer PZT signal in anesthetized animals in our setting. Our experimental approach is therefore not capable of measuring e.g. tidal volumes. It is known that thorax movements strongly relate to phrenic nerve activity and are therefore an indicator of breathing frequency [10], [29]. However, we validated our finding on reduced breathing frequency upon urethane treatment by evaluation of blood gas analyses, which confirmed a hypoventilatory effect of urethane. We observed that urethane at 1 g per kg body weight leads to a significant reduction in respiration rate by about 33% in rats and 15% in mice at P6/7. This hypoventilation in both species significantly affected blood gases and pH. Analyses of blood gases in P6/7 mice and rats confirmed the reduction of ventilation upon treatment with urethane as reflected by an increase in pCO_2_. This indicates that during anesthesia the ability to cope with alterations in ventilation may be significantly perturbed in newborn C57/Bl6 mice and Wistar rats_._ Interestingly we observed a reduction in RPM upon urethane administration in all cases except for P0/1 rats. To elucidate if this discrepancy may be due to differences in pharmacokinetics of urethane at P0/1 we assessed ethanol concentrations in plasma of urethane treated animals. One of the major metabolites of urethane by hydrolysis in plasma is ethanol [17]. Our results on ethanol levels in blood plasma from mice and rats at P0/1 and P6/7 show that urethane pharmacokinetics are different between P0/1 and P6/7. Significantly higher ethanol concentrations found in plasma of P0/1 mice compared with P0/1 rats indicate that urethane metabolism is different between P0/1 mice and rats. In contrast ethanol in plasma was not present at P6/7 in both species. Our data demonstrate that the metabolism of urethane is highly variable depending on age and species in the first postnatal week. These discrepancies may hold responsible for the absence of a hypoventilation after urethane administration in P0/1 rats. However the exact mechanism for this observation is unclear. It has been demonstrated that in adult mice cytochrome P450 enzymes are another major pathway for urethane metabolism [30]. We therefore speculate that beside differences in urethane metabolism by hydrolysis a different activity or expression of enzymes located in the liver of P0/1 mice and rats may also hold responsible for the different effect of urethane on breathing frequency at this age. More pharmacologic research dealing with urethane metabolism in neonatal rodents needs to be done to resolve this question in detail.

It has been documented in rodents that urethane, depending on its dosage, has moderate effects on heart rate [31]–[33]. De Wildt et al. demonstrated that urethane at a dosage of 1 g per kg body weight does not affect heart frequency in adult rats [33]. In line with these observations we noted that heart rate was not affected in neonates by urethane treatment (1 g per kg body weight). Field and coworkers documented a decrease of pH upon urethane administration at a dose of 1.2–1.5 g/kg body weight in male rats [31]. Based on these reports and our data we conclude that in newborn mice and rats, urethane results in an acidosis as previously reported in adult rats [31]. However, Field and colleagues reported a hyperventilatory effect of urethane in adult rats which is in contrast to our recordings in neonates. In our study urethane induced a hypoventilation in mice and rat pups reflected by a decrease of RPM and an increase in pCO_2_ in blood gas analyses. Further we observed a reduced level of pH in anesthetized animals whilst base excess and bicarbonate concentration were not different to animals that received sham injections. These findings are in agreement with a respiratory non-compensated acidosis [34] upon urethane within the first 30–60 minutes after injection.

One explanation for the observed discrepancy to the data by Field may be the lower dose of 1 g per kg bodyweight in our study and genetic differences in inbred strains and the different age of animals. Further one may speculate that at later time points during urethane anesthesia a switch from primary respiratory acidosis to a metabolic acidosis takes place that ultimately results in compensatory hyperventilation.

Our results indicate that the reduction in RPM in newborn mice and rats by urethane is more prevalent in P0/1 mice, absent in P0/1 rats but present in both species at P6/7. We confirmed that the reduction in RPM reflected by reduced thoracic or abdominal movement causes hypoventilation by analyzing blood gases. Here pCO_2_ was significantly elevated in urethane treated animals and resulted in a respiratory acidosis.

Hypercapnia in newborn piglets has been shown to alter neuronal function in the cerebral cortex [35]. CO_2_ is a potent vasodilator in brain vessels at high concentration while it results in vasoconstriction in cerebral vessels at low pCO_2_ levels [36]. These data indicate that changes in pCO_2_ and pH may introduce experimental errors. With regard to changes in electrophysiological recordings upon urethane treatment we have previously shown that urethane specifically affects the amplitude of gamma oscillations while other patterns like spindle bursts and long oscillations are not affected [8]. Based on our results regarding the effect of urethane on ventilation and blood gases in newborn rodents we recommend to monitor cardio-respiratory parameters when using urethane as anesthetic agent.

### Conclusion

We present a simple setup consisting of a piezoelectric transducer element and a two channel ECG, which allows monitoring of cardio-respiratory parameters in anesthetized and awake newborn mice and rats. Our data demonstrate the relevance of monitoring cardio-respiratory parameters during *in vivo* investigations. To avoid experimental errors, care should be taken to maintain cardio-respiratory parameters in neonates within a physiological range.

## References

[1] Attwell D, Buchan AM, Charpak S, Lauritzen M, MacVicar BA, et al (2010) Glial and neuronal control of brain blood flow. Nature 468: 232–243 doi:10.1038/nature09613. 21068832 10.1038/nature09613 PMC3206737

[2] Franceschini MA, Radhakrishnan H, Thakur K, Wu W, Ruvinskaya S, et al (2010) The effect of different anesthetics on neurovascular coupling. Neuroimage 51: 1367–1377 doi:10.1016/j.neuroimage.2010.03.060. 20350606 10.1016/j.neuroimage.2010.03.060 PMC2879067

[3] Masamoto K, Kanno I (2012) Anesthesia and the quantitative evaluation of neurovascular coupling. Journal of cerebral blood flow and metabolism: official journal of the International Society of Cerebral Blood Flow and Metabolism. Available: http://www.ncbi.nlm.nih.gov/pubmed/22510601. Accessed 19 June 2012. 10.1038/jcbfm.2012.50 PMC3390804 22510601

[4] Lu H, Werner C, Engelhard K, Scholz M, Kochs E (1998) The effects of sevoflurane on cerebral blood flow autoregulation in rats. Anesth Analg 87: 854–858. 9768782 10.1097/00000539-199810000-00020

[5] Tétrault S, Chever O, Sik A, Amzica F (2008) Opening of the blood-brain barrier during isoflurane anaesthesia. Eur J Neurosci 28: 1330–1341 doi:10.1111/j.1460-9568.2008.06443.x. 18973560 10.1111/j.1460-9568.2008.06443.x

[6] Thal SC, Luh C, Schaible E-V, Timaru-Kast R, Hedrich J, et al (2012) Volatile Anesthetics Influence Blood-Brain Barrier Integrity by Modulation of Tight Junction Protein Expression in Traumatic Brain Injury. PLoS ONE 7: e50752 doi:10.1371/journal.pone.0050752. 23251381 10.1371/journal.pone.0050752 PMC3519465

[7] Pagliardini S, Greer JJ, Funk GD, Dickson CT (2012) State-dependent modulation of breathing in urethane-anesthetized rats. J Neurosci 32: 11259–11270 doi:10.1523/JNEUROSCI.0948-12.2012. 22895710 10.1523/JNEUROSCI.0948-12.2012 PMC6621193

[8] Yang J-W, Hanganu-Opatz IL, Sun J-J, Luhmann HJ (2009) Three patterns of oscillatory activity differentially synchronize developing neocortical networks in vivo. J Neurosci 29: 9011–9025 doi:10.1523/JNEUROSCI.5646-08.2009. 19605639 10.1523/JNEUROSCI.5646-08.2009 PMC6665441

[9] Favrais G, Van de Looij Y, Fleiss B, Ramanantsoa N, Bonnin P, et al (2011) Systemic inflammation disrupts the developmental program of white matter. Annals of Neurology 70: 550–565 doi:10.1002/ana.22489. 21796662 10.1002/ana.22489

[10] Robinson DM, Kwok H, Adams BM, Peebles KC, Funk GD (2000) Development of the ventilatory response to hypoxia in Swiss CD-1 mice. J Appl Physiol 88: 1907–1914. 10797156 10.1152/jappl.2000.88.5.1907

[11] Matrot B (2004) Automatic classification of activity and apneas using whole body plethysmography in newborn mice. Journal of Applied Physiology 98: 365–370 doi:10.1152/japplphysiol.00803.2004. 10.1152/japplphysiol.00803.2004 15591306

[12] Ramanantsoa N, Vaubourg V, Matrot B, Vardon G, Dauger S, et al (2007) Effects of temperature on ventilatory response to hypercapnia in newborn mice heterozygous for transcription factor Phox2b. AJP: Regulatory, Integrative and Comparative Physiology 293: R2027–R2035 doi:10.1152/ajpregu.00349.2007. 10.1152/ajpregu.00349.2007 17715184

[13] An S, Yang J-W, Sun H, Kilb W, Luhmann HJ (2012) Long-term potentiation in the neonatal rat barrel cortex in vivo. J Neurosci 32: 9511–9516 doi:10.1523/JNEUROSCI.1212-12.2012. 22787036 10.1523/JNEUROSCI.1212-12.2012 PMC6622258

[14] Brockmann MD, Pöschel B, Cichon N, Hanganu-Opatz IL (2011) Coupled Oscillations Mediate Directed Interactions between Prefrontal Cortex and Hippocampus of the Neonatal Rat. Neuron 71: 332–347 doi:10.1016/j.neuron.2011.05.041. 21791291 10.1016/j.neuron.2011.05.041

[15] Paton JF, Richter DW (1995) Maturational changes in the respiratory rhythm generator of the mouse. Pflugers Arch 430: 115–124. 7667071 10.1007/BF00373846

[16] Schmidt RF, Lang F, Heckmann M (2011) Physiologie des Menschen mit Pathophysiologie. Berlin, Heidelberg: Springer-Verlag Berlin Heidelberg. Available: http://dx.doi.org/10.1007/978-3-642-01651-6. Accessed 26 November 2012.

[17] Salmon AG, Zeise L (1991) Risks of carcinogenesis from urethane exposure. Boca Raton, Fla: CRC Press. 231 p.

[18] Bartels H, Bartels R, Rathschlag-Schaefer AM, Röbbel H, Lüdders S (1979) Acclimatization of newborn rats and guinea pigs to 3000 to 5000 m simulated altitudes. Respir Physiol 36: 375–389. 441588 10.1016/0034-5687(79)90049-5

[19] Hou PC, Burggren WW (1989) Interaction of allometry and development in the mouse Mus musculus: heart rate and hematology. Respir Physiol 78: 265–280. 2616924 10.1016/0034-5687(89)90103-5

[20] Teppema LJ, Dahan A (2010) The ventilatory response to hypoxia in mammals: mechanisms, measurement, and analysis. Physiol Rev 90: 675–754 doi:10.1152/physrev.00012.2009. 20393196 10.1152/physrev.00012.2009

[21] Mortola JP, Rezzonico R, Lanthier C (1989) Ventilation and oxygen consumption during acute hypoxia in newborn mammals: a comparative analysis. Respir Physiol 78: 31–43. 2813986 10.1016/0034-5687(89)90140-0

[22] Bleich HL, Moore MJ, Cryer PE (1980) Physiology and Pathophysiology of the Human Sympathoadrenal Neuroendocrine System. New England Journal of Medicine 303: 436–444 doi:10.1056/NEJM198008213030806. 6248784 10.1056/NEJM198008213030806

[23] Sato S (2008) Quantitative evaluation of ontogenetic change in heart rate and its autonomic regulation in newborn mice with the use of a noninvasive piezoelectric sensor. AJP: Heart and Circulatory Physiology 294: H1708–H1715 doi:10.1152/ajpheart.01122.2007. 18263713 10.1152/ajpheart.01122.2007

[24] Sato S, Yamada K, Inagaki N (2006) System for simultaneously monitoring heart and breathing rate in mice using a piezoelectric transducer. Med Biol Eng Comput 44: 353–362 doi:10.1007/s11517-006-0047-z. 16937177 10.1007/s11517-006-0047-z

[25] Cabot RC, Harris NL, Shepard J-AO, Rosenberg ES, Cort AM, et al (2012) Case 19-2012. New England Journal of Medicine 366: 2409–2419 doi:10.1056/NEJMcpc1109276. 22716980 10.1056/NEJMcpc1109276

[26] Gootman PM, Buckley BJ, DiRusso SM, Gootman N, Yao AC, et al (1986) Age-related responses to stimulation of cardiopulmonary receptors in swine. Am J Physiol 251: H748–755. 3766752 10.1152/ajpheart.1986.251.4.H748

[27] Downing SE, Lee JC, Taylor JF (1977) Cardiac function and metabolism during cholinergic stimulation in the newborn lamb. Am J Physiol 233: H451–457. 910963 10.1152/ajpheart.1977.233.4.H451

[28] Tankersley CG, Fitzgerald RS, Levitt RC, Mitzner WA, Ewart SL, et al (1997) Genetic control of differential baseline breathing pattern. J Appl Physiol 82: 874–881. 9074977 10.1152/jappl.1997.82.3.874

[29] Hilaire G, Bou C, Monteau R (1997) Rostral ventrolateral medulla and respiratory rhythmogenesis in mice. Neurosci Lett 224: 13–16. 9132679 10.1016/s0304-3940(97)13458-9

[30] Hoffler U (2003) Cytochrome P450 2E1 (CYP2E1) Is the Principal Enzyme Responsible for Urethane Metabolism: Comparative Studies Using CYP2E1-Null and Wild-Type Mice. Journal of Pharmacology and Experimental Therapeutics 305: 557–564 doi:10.1124/jpet.103.049072. 12704224 10.1124/jpet.102.049072

[31] Field KJ, White WJ, Lang CM (1993) Anaesthetic effects of chloral hydrate, pentobarbitone and urethane in adult male rats. Lab Anim 27: 258–269. 8366672 10.1258/002367793780745471

[32] Maggi CA, Meli A (1986) Suitability of urethane anesthesia for physiopharmacological investigations in various systems. Part 2: Cardiovascular system. Experientia 42: 292–297. 3007197 10.1007/BF01942510

[33] De Wildt DJ, Hillen FC, Rauws AG, Sangster B (1983) Etomidate-anaesthesia, with and without fentanyl, compared with urethane-anaesthesia in the rat. Br J Pharmacol 79: 461–469. 6652337 10.1111/j.1476-5381.1983.tb11019.x PMC2044874

[34] Ayers P, Warrington L (2008) Diagnosis and treatment of simple acid-base disorders. Nutr Clin Pract 23: 122–127 doi:10.1177/0884533608314534. 18390779 10.1177/0884533608314534

[35] Fritz KI, Zubrow A, Mishra OP, Delivoria-Papadopoulos M (2005) Hypercapnia-Induced Modifications of Neuronal Function in the Cerebral Cortex of Newborn Piglets. Pediatric Research 57: 299–304 doi:10.1203/01.PDR.0000148718.47137.9B. 15585683 10.1203/01.PDR.0000148718.47137.9B

[36] Willie CK, Macleod DB, Shaw AD, Smith KJ, Tzeng YC, et al (2012) Regional brain blood flow in man during acute changes in arterial blood gases. J Physiol (Lond) 590: 3261–3275 doi:10.1113/jphysiol.2012.228551. 22495584 10.1113/jphysiol.2012.228551 PMC3459041

